# To asses functional out-come of distal femur fracture treated with distal femur locking plate using American knee society score

**DOI:** 10.6026/973206300220889

**Published:** 2026-02-28

**Authors:** Amol Kawde, Sanjul Bansal, Kartikeya Singh, Aayush Kumar Soni, Shrajay Singh Rawat, Pratima Nagale, Abhishek Keshav, Sachin Parmar

**Affiliations:** 1 Department of Orthopaedics, ABVGMC, Vidisha, Madhya Pradesh, India; 2 Department of Orthopaedics, LNCT Medical College and Sevakunj Hospital, Indore, Madhya Pradesh, India; 3 Department of Orthopaedics, RKMC, Bhopal, Madhya Pradesh, India; 4 Department of Orthopaedics, SAMC and PGI, Indore, Madhya Pradesh, India; 5 Department of Emergency Medicine, ABVGMC, Vidisha, Madhya Pradesh, India; 6 Department of Orthopaedics, AIIMS, Bhopal, Madhya Pradesh, India; 7 Department of Community Medicine, V.K.S. Government Medical College, Neemuch, Madhya Pradesh India

**Keywords:** Distal femur fracture, distal femur locking plate, American Knee Society Score (AKSS), AO/ASIF classification, functional outcome

## Abstract

Despite advances in fixation techniques, distal femur fractures treated with distal femur locking plates can have inconsistent functional recovery; therefore, functional outcome assessment using the American Knee Society Score (AKSS) is required. Locking plates on the distal femur provide greater stability and resistance to reduction in alignment during the management of distal femoral fractures when designs with fixed angles proved insufficient to manage some types of fractures. In this research, which was carried out at Sri Aurobindo Medical College and PG Institute, the authors assessed the results of 30 patients with closed distal femur fracture treated with these plates. The results showed good to excellent functional outcomes, 56.7 percent of patients had excellent results and few complications such as shortening of limbs (6.7) and infection (10). Thus, we show that locking compression plates are useful in the treatment of fractures of the distal femur.

## Background:

Distal femur locking plates have proven effective in treating distal femoral fractures by offering enhanced stability and improved protection against both initial and subsequent reductions in alignment [1, 2, 3, 4, 5, 6, 7, 8- 9]. The initial iteration of locked plates featured threaded holes with a fixed angle, providing stable fixation around the joint. However, all the holes in the plate held the screws at the identical angle. This fixed angle proved unfavorable for certain types of fractures in the lower end of the thigh bone, such as when inserting screws around artificial joints in fractures near the joint [10, 11, 12- 13]. In order to reduce these issues, various locking plates have been developed, expanding on the idea of screws used in fixation systems [14]. These plates offer a wide range of possibilities for adjusting the angle of the screws, which improves the ability to treat fractures. This adaptability enables a secure fixation of extensively fragmented or weakened fractures. Multiple technologies are available for achieving variable axis locking, such as self-locking bushings or two-component screws where a cap secures the screw in a specific direction. The reliability of the plate in biomechanical performance is a significant concern. Recent researches indicate that there is no substantial disparity observed between standard plates and locking plates in terms of failure at the screw and plate interface [15]. There have been a limited number of clinical publications [12] on the use of distal femur plate implants. Early observations indicate that these implants operate well and have complication rates similar to the first-generation plates. Therefore, it is of interest to provide AKSS-based functional outcome data for distal femur fractures managed with distal femur locking plates in the local/your institutional setting, helping benchmark results against other series.

## Materials and Methods:

Cross sectional done in orthopaedic department of Sri Aurobindo Medical College and PG Institute, Indore. Patient with distal femur fracture gets himself/herself investigated, including X-Ray, routine OT profile etc., Patients are assessed using American knee society score during pre-operative period and post-operative period by regular follow-ups at 6 weeks, 3 month and, 6 month. patient Age >18 YEARS with closed distal femur fracture were included while patients with Open fracture, Patient with fracture of other bone of same limb, Patient not giving consent and fractures associated with neurovascular injuries were excluded. An informed written consent will be taken from all the patients after the approval of institutional ethical committee. Finally after the diagnosis, the patients are selected for the study depending on the inclusion criteria. After obtaining a detailed history, complete general physical and systemic examination, the patients will be subjected to relevant investigations. The complete data will be recorded in a specially designed case recording form. The data will be recorded on the pre-structured proforma especially designed for the study. History by verbal communication with patients and their attendants, Clinical examination, both local and systemic, Basic Radiological Examination.

## Diagnosis:

Clinical and Radiological, Base line investigation, Post-Operative evaluation by clinical examination and radiographs and any post-operative complications. The data will be collected and entered in MS excel. Data will be presented as frequency table. Descriptive statistics will be calculated for quantitative variable (mean & SD) and categorical variable (frequency & percentage).

## Results:

Among 30 patients, most were aged 21-40 years (56.7%), followed by 41-60 years (33.3%) and 18-20 years (10.0%), with a clear male predominance (83.3% males vs 16.7% females) (Table 1). Road traffic accidents were the principal mechanism of injury (90.0%), while falls (6.7%) and assault (3.3%) were uncommon (Table 2). As per AO/ASIF classification, C-type fractures were most frequent (C1 23.3%, C2 16.7%, C3 16.7%), followed by A2 (20.0%), A1 (13.3%), A3 (6.7%) and B2 (3.3%) (Table 3). Radiological union was achieved most commonly within 19-20 weeks (43.3%), followed by 16-18 weeks (26.7%), 21-22 weeks (20.0%), while 10.0% required >22 weeks (Table 4). At final assessment, knee flexion was ≥110°C in 43.3%, 91-109°C in 30.0% and <90°C in 26.7%, while knee extensor lag was minimal (0-5°C) in 96.7% of cases (Table 5). Functional outcomes were predominantly favorable, with excellent results in 56.7% and good in 33.3% by Neer's scoring (Table 6) and by AKSS most patients achieved good (43.33%) or excellent (20.0%) scores though fair (23.33%) and poor (13.34%) outcomes were also noted (Table 7). Complications were infrequent, with 83.3% reporting none; infection occurred in 10.0% and limb shortening (10 mm) in 6.7% (Table 8).

## Discussion:

Fractures occurring at the lower end of the femur have a prevalence rate of 1% among all types of fractures and account for 4-6% of all femoral fractures [16, 17 and 18]. Due to the high frequency of complications associated with these fractures, orthopaedic surgeons face a challenging task in treating them. The locking compression plate offers advantages such as several sites of fixed plate to screw contact, which enhances stability through a single lateral design. Due to the reduced probability of screw loosening, this also results in a more robust fixation. The majority of patients fell between the age ranges of 21-40 years, with an average age of 37.87±11.76 years (ranging from 18 to 58 years). There was a higher number of men (25) compared to females (5), indicating male predominance. The degree of participation on the right side was more noticeable as compared to the left side.

In their study, Kumar *et al.* included patients with a mean age of 35 years (ranging from 20 to 72 years), consisting of 36 males and 10 females [19]. The patients in the study conducted by Mahesh had a mean age of 53 years, with an age range of 20 to 86 years. There were 7 male patients and 3.8 female patients [20]. The average age of our patients is similar to the study conducted by Kumar, however the studies conducted by Mahesh, Pipal and Reddy have revealed a greater average age. Road traffic accidents were the primary source of damage observed in 90% of the patients. Based on the AO/ASIF classification, type C1 was the most prevalent (23.3%), followed bytypeA2 (20%) and type C2 and C3, each observed in 5 patients (16.7%). Fifteen (50%) individuals exhibited concomitant bony injuries. In their study, Kadam *et al.* reported a 75% rate of road traffic accidents. Garg & Garg also documented that road traffic accidents are the most prevalent cause of injury [21, 22]. Poptani & Lonikar also found that 75% of injuries were from road traffic incidents [23]. Out of the total number of patients, 43.3% obtained a knee flexion greater than 110 degrees, 30% had a knee flexion between 91 and 109 degrees and 26.7% had a knee flexion less than 90 degrees. In their study, Kadam *et al.* found that 50% of the patients exhibited knee flexion of 110 degrees, whereas8patientshad arranges of 91 to 109 degrees and 2 patients (10%) had knee flexion less than 90 degrees. In our investigation, the prevalence of knee flexion less than 90 degrees was slightly higher compared to the Kadam study [21]. Each patient had an extensor lag of less than 5 degrees, except for one (3.3%) who had one of 10 degrees or more. In their study, Poptani and Lonikar found that the average extender lag was 5.6 degrees [23]. In their work, Mani *et al.* found an extensor lag of over 15 degrees [24]. Our results are similar to those of Poptani's work, but Mani found a bigger extensor lag.

## Conclusion:

The utilization of a locking compression plate for the treatment of a fracture at the lower end of the femur has been observed to yield favourable to outstanding functional results in most patients, with a minimal occurrence of sequelae. The complications were easily controllable. The only problems that were encountered were limb shortening and infection. We strongly recommend using a locking compression plate for the treatment of a distal end femur fracture.

## Figures and Tables

**Table 1:** Distribution of patients according to age and sex30 patients

**Particular**	**N**	**Percentage (%)**
**Age group (years)**		
18-20 years	3	10
21-40 years	17	56.7
41-60 years	10	33.3
Total	30	100
**Sex**		
Female	5	16.7
Male	25	83.3
Total	30	100

**Table 2:** Distribution of patients according to mechanism of injury

**Mechanism of injury**	**N**	**Percentage (%)**
Assault	1	3.3
Fall	2	6.7
Road traffic accident	27	90
Total	30	100

**Table 3:** Distribution of patients according to type of fracture(AO/ASIF classification system)

**Type of fracture**	**N**	**Percentage (%)**
A1	4	13.3
A2	6	20
A3	2	6.7
B2	1	3.3
C1	7	23.3
C2	5	16.7
C3	5	16.7
Total	30	100

**Table 4:** Distribution of patients according to radiological union time

**Radiological union time (weeks)**	**N**	**Percentage (%)**
16-18	8	26.7
19-20	13	43.3
21-22	6	20
>22	3	10
Total	30	100

**Table 5:** Distribution according to knee flexion and knee extensor lag

**Variables**	**N**	**Percentage (%)**
**Knee flexion**		
<90 degrees	8	26.7
91-109degrees	9	30
≥110 degrees	13	43.3
Total	30	100
**Knee extensor lag**		
0-5 degrees	29	96.7
6-10 degrees	1	3.3
>10 degrees	-	-
Total	30	100

**Table 6:** Distribution according to functional outcome as per Neersscoring

**Functional outcome**	**N**	**Percentage (%)**
Poor	2	6.7
Fair	1	3.3
Good	10	33.3
Excellent	17	56.7
Total	30	100

**Table 7:** Distribution according to functional outcome as per AKSS Outcome Score

**AKSS Outcome Score**	**Frequency**	**Percentage (%)**
Excellent	6	20
Good	13	43.33
Fair	7	23.33
Poor	4	13.34

**Table 8:** Distribution according to complications

**Complications**	**N**	**Percentage (%)**
Limb shortening (10mm)	2	6.7
Infection	3	10
None	25	83.3
Total	30	100
